# Role of Transient Receptor Potential Vanilloid 1 in Sonic Hedgehog-Dependent Taste Bud Differentiation

**DOI:** 10.3390/life13010075

**Published:** 2022-12-27

**Authors:** Yun-Hee Rhee, Young-Hoon Choi, Allison C. Hu, Min Young Lee, Jin-Chul Ahn, Sehwan Kim, Ji-Hun Mo, Seung Hoon Woo, Phil-Sang Chung

**Affiliations:** 1 Beckman Laser Institute Korea, Dankook University, 119 Dandae-ro, Cheonan 31116, Republic of Korea; 2 Medical Laser Research Center, Dankook University, 119 Dandae-ro, Cheonan 31116, Republic of Korea; 3 Laser Translational Clinical Trial Center, Dankook University Hospital, Cheonan 31116, Republic of Korea; 4 Beckman Laser Institute and Medical Clinic, University of California Irvine, 1002 Health Sciences Rd., Irvine, CA 92697, USA; 5 Department of Otolaryngology-Head and Neck Surgery, College of Medicine, Dankook University, Cheonan 31116, Republic of Korea

**Keywords:** taste bud cells, innervation, transient receptor potential vanilloid 1, sonic hedgehog

## Abstract

Taste bud cell differentiation is extremely important for taste sensation. Immature taste bud cells cannot function during taste perception transmission to the nerve. In this study, we investigated whether hedgehog signaling affected taste bud cell differentiation and whether transient receptor potential vanilloid 1 (TRPV1) played a key role in dry mouth. The induction of dry mouth due to salivary gland resection (SGR) was confirmed on the basis of reduced salivation and disrupted fungiform papillae. The expression of keratin 8 (K8) of taste bud cells, neurofilament (NF), sonic hedgehog (Shh), and glioma-associated oncogene homolog 1 (Gli1) around taste bud cells was downregulated; however, the expression of TRPV1, P2X purinoceptor 3 (P2X3), and hematopoietic stem cell factor (c-Kit) was upregulated at the NF ends in the dry mouth group. To investigate the effect of TRPV1 defect on dry mouth, we induced dry mouth in the TRPV-/- group. The K8, NF, and P2X3 expression patterns were the same in the TRPV1 wild-type and TRPV1-/- dry mouth groups. However, Shh and c-Kit expression decreased regardless of dry mouth in the case of TRPV1 deficiency. These results indicated that TRPV1 positively regulated proliferation during taste bud cell injury by blocking the Shh/Gli1 pathway. In addition, not only cell proliferation but also differentiation of taste bud cells could not be regulated under TRPV1-deficiency conditions. Thus, TRPV1 positively regulates taste bud cell innervation and differentiation; this finding could be valuable in the clinical treatment of dry mouth-related taste dysfunction.

## 1. Introduction

Dry mouth is a severe disease condition that disrupts homeostasis in the gustatory system and is caused by factors such as chemotherapy [1] and aging [2]. Dry mouth exacerbates dental caries and periodontal disease [3] and impairs mastication, swallowing, and sleeping. It also causes burning mouth syndrome and dysgeusia [4,5], thus severely impairing the quality of life of patients. Various chemical methods for treating dry mouth have been reported [6,7,8]; these methods are not based on molecular targets associated with taste bud innervation.

Taste buds contain neuroepithelial, neuroendocrine, neuromodulator, and chemoreceptor cells [9,10,11,12] since they have varying levels of epithelial, neuronal, and secretory properties. In addition, the distinctive cellular and molecular characteristics of taste bud cells suggest the presence of multiple stem cell types [13,14]. Impairment of parasympathetic innervation has been reported to decelerate progenitor cell differentiation in nerves [15,16]. Innervations are essential for gustation and taste bud cell maintenance, and hedgehog/glioma-associated oncogene homolog 1 (Gli) signaling plays a crucial role in this process [17,18,19,20]. Recent studies have reported that transient receptor potential vanilloid 1 (TRPV1) expression influences both postnatal and activity-induced neurogenesis during adulthood [21,22,23]. For example, neuronal TRPV1 promoted the release of bioactive neuropeptides from sensory fiber innervation organs [21,22], and TRPV1-immunoreactivity was detected in nerve fibers beneath the epithelium, and/or taste bud-like structure [23,24]. However, the role of TRPV1 in restoring taste bud function in individuals with dry mouth and its interaction with hedgehog/Gli signaling remain unclear.

Therefore, in this study, we aimed to determine whether dry mouth influences taste bud maintenance and evaluate the effect of dry mouth on nerve/stem cell signaling in an animal model of dry mouth syndrome. Furthermore, we evaluated the role played by TRPV1 in innervation during taste bud differentiation by using TRPV1-knockout mice.

## 2. Materials and Methods

### 2.1. Animals

Eight-week-old C57BL6 mice were purchased from Orient Bio (Suwon, Republic of Korea). All the animal procedures used in the current study were performed in accordance with the Institutional Animal Care and Use Committee (IACUC) guidelines and were approved by the Ethics Committee of Dankook University (DKU 17-006). TRPV1-knockout mice (Strain: B6.129X1-Trpvqtm1Jul) were purchased from The Jackson Laboratory (Bar Harbor, ME, USA), and the strain was maintained via genotyping analysis. The primers and polymerase chain reaction (PCR) protocol used have been described in the Supplementary Data (Figure S1).

### 2.2. Experimental Protocol

**Figure 1:**
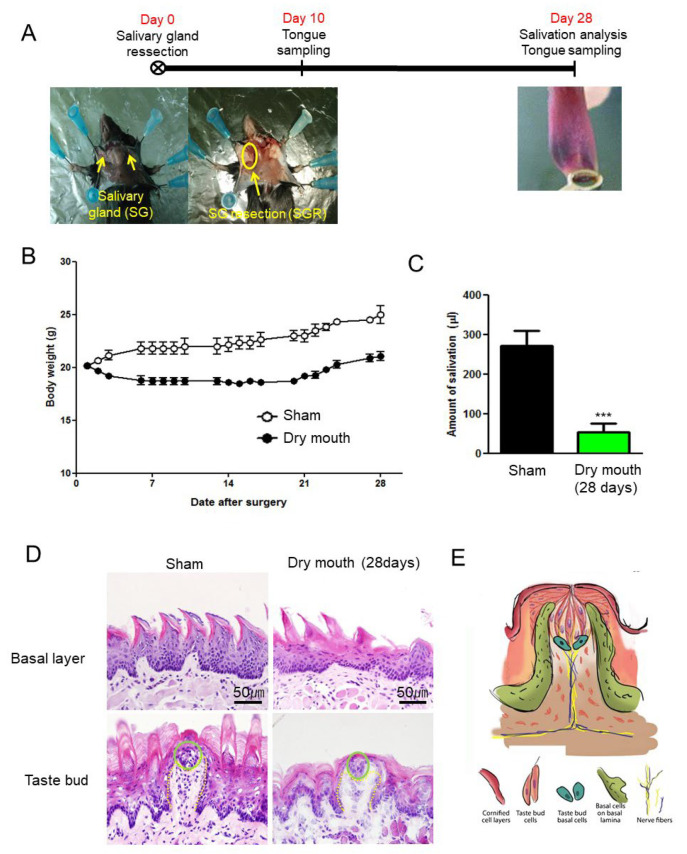
Validation of induction of dry mouth after salivary gland resection (SGR). (**A**) The wild-type and TRPV1-knockout mice were randomly distributed into two groups of eight animals each: the sham and SGR groups. The salivary glands of the mice were removed in the SGR group. To exclude the effects of incision and anesthesia, the sham group was performed only after incision. (**B**) After surgery, the body weight of the mice was measured for 5 weeks. The body weight of the SGR group mice was not higher than that of the sham group mice. (**C**) The saliva volume of the mice was measured at 4 weeks after surgery. To induce salivation in each mouse, 0.3 mg/kg pilocarpine was intraperitoneally administered at 3 min after the mice were anesthetized. The secreted saliva was collected with natural flow into tubes for 10 min. The values have been provided in terms of mean ± standard error of mean (SEM). *** *p* < 0.001. (**D**) The anterior tongue was removed at 10 days after SGR. Subsequently, H&E staining demonstrated that the morphological features of the basal layers and taste buds (green circle) were disrupted. The dashed lines demarcate the epithelial border, and the scale bar provided applies to all the images. (**E**) Diagram of the taste bud in fungiform papillae. The fungiform papillae in the mouse accommodate one apical taste bud and are innervated by lingual and taste bud-specific chorda tympani nerves.

Sixteen wild-type and TRPV1-knockout mice were randomly distributed into two groups of eight mice each: the sham and salivary gland resection (SGR) groups. The mice were anesthetized via intraperitoneal injection of 10 mg/kg Zoletil (Verbac, France) and 5 mg/kg Rompun (Bayer, Korea), and the salivary glands were resected only in the SGR group. To exclude the effects of incision and anesthesia, the sham group was subjected to a sham operation that only involved an incision. After surgery, the body weight and saliva volume of the mice were measured for 28 days (Figure 1A). To induce salivation in the mice, 0.3 mg/kg pilocarpine (Sigma, St. Lois, MO, USA) was intraperitoneally administered at 3 min after anesthesia [25]. The secreted saliva was collected in tubes for 10 min. The volume of secreted saliva was measured using a 100 µL pipette. We sampled the tongue at 10 days after SGR because the tongue epithelium undergoes continual turnover, being replenished every 3–5 days in mice, and the life spans of short- and long-lived taste bud cells are usually approximately 2–21 days or more. The anterior tongue of the mice from each group was retrieved en bloc after live imaging of mouse tongue observation by two-photon microscope and stored at −80 °C after embedding in optimal cutting temperature (OCT) compound.

### 2.3. Chemicals and Antibodies

Rabbit polyclonal anti-keratin 8 (K8) antibody (TROMA-1) was purchased from Developmental Studies Hybridoma Bank (Iowa City, IA, USA), and rabbit polyclonal anti-c-Kit antibody was purchased from Cell Signaling (Beverly, MA, USA). Rabbit polyclonal anti-TRPV1 (VR1), anti-Gli1, and anti–sonic hedgehog (SHH) antibodies were purchased from Abcam (Cambridge, MA, USA). Rabbit polyclonal anti-P2X3 antibody was purchased from Novus Bio (Littleton, CO, USA), and donkey polyclonal anti-neurofilament (NF) antibody was purchased from Millipore (Temecula, CA, USA). Alexa 488 and Alexa 657 were purchased from Life Technologies (Grand Island, NY, USA). Calcium green-1 dextran, potassium salt was purchased from ThermoFisher Scientific (Waltham, MA, USA). OCT compound was purchased from Sakura Fine Technical (Tokyo, Japan), bovine serum albumin (BSA) was purchased from Santa Cruz Biotechnology (Santa Cruz, CA, USA), and 4′,6-diamidino-2-phenylindole (DAPI) fluorescence mount was purchased from Vector (Burlingame, CA, USA).

### 2.4. Live Imaging of Mouse Tongue by Two-Photon Microscope

We operated customized stainless-steel metal plates which could hold mouse tongue without any damage during live imaging, referring to Choi et al.’s work [26]. The externalized tongue was sandwiched between two metals. There is a hole in the upper plate that allows the objective lens to capture live images of the mouse tongue. The space between the objective lens and mouse tongue was immersed with water under upper plate. A water immersion objective (16X, Olympus) was equipped with a home-built two-photon microscope (Olympus BX74), 800–810 nm wavelength used for the two-photon excitation (Coherent, Chameleon 21 Vision II). To visualize the calcium reaction in the taste buds, 50 mM of an anionic calcium indicator (calcium green-1 dextran) was loaded onto the tongue dorsum using tweezer-type electrode (5 Vpp, 2 Hz, pulse width: 100 ms, duration: 10 s) and rinsed using phosphate-buffered saline (PBS) (Supplementary Figure S2. After repeating this process 2–3 times, 3 μM capsaicin was administered and live images of calcium activity was observed. Fluorescent images were captured at ~6 Hz with emissions at 525 ± 50 nm for anionic calcium indicator. Using 4 mice for each group, live images were taken twice for 45 s, and comparative quantification was calculated from the average of the two images of four mice.

### 2.5. Histological Analysis

The blocks were sliced at 8 mm thickness by using a microtome (Leica, Buffalo Grove, IL, USA), and the tissues were fixed with acetone. The samples were washed with PBS and stained in Harris hematoxylin and eosin Y solution. Subsequently, the tissue sections were observed using a microscope (Olympus BX51; Miami, FL, USA).

### 2.6. Immunofluorescence Analysis

The tissue samples were subjected to conventional histological preparation and blocked with 1% goat serum in PBS for 1 h. Double staining was performed using red and green fluorescence. After the blocking step, the tissue samples were probed with primary antibodies at 4 °C overnight. Subsequently, the tissue samples were probed with Alexa 488 (green)- and Alexa 657 (red)-conjugated secondary antibodies for 2 h, following which they were carefully washed with PBS. For nuclear staining, the samples were mounted using fluorescence mount solution with DAPI. After the samples were stained, they were observed using a confocal microscope (FV3000; Olympus, Tokyo, Japan), and the staining intensity was measured and converted to relative fluorescence units (RFUs) by using the FV3000 software program. Protein expression was quantified using ImageJ (NIH, Bethesda, MD, USA).

### 2.7. Statistical Analysis

The number of mice used in the analysis is indicated by n in the figures. Taste bud-containing areas were randomly selected in three specimens from each mouse and photographed under 40× magnification. Live imaging comparative data have been expressed in terms of means ± standard deviation (SD) values. Differences between the sham and SGR groups were determined using Tukey *t*-test with one-way ANOVA by using Prism^®^ (GraphPad, La Jolla, CA, USA). Quantitative data have been expressed in terms of mean ± standard error of mean (SEM) values. Differences were determined using an unpaired *t*-test with Welch’s correction by using Prism^®^. Statistical significance was determined at * *p* < 0.1, ** *p* < 0.05, and *** *p* < 0.001, and the F test was used to compare variances.

## 3. Results

### 3.1. SGR Induced Body Weight Decrease and Dry Mouth

To confirm that SGR induced dry mouth, we measured the body weight and salivary secretion after SGR. The body weight differed between the sham and dry mouth groups after SGR (Figure 1B). The body weight increase noted at 28 days after SGR in the dry mouth group (0.9 ± 0.6 g from baseline) was significantly lower than that in the sham group (4.8 ± 1.3 g from baseline; n = 8; *p* < 0.001, F = 23.61). The salivary volume of the dry mouth group (53 ± 18.9 μL; n = 6) was significantly lower than that of the sham group (271 ± 38.4 μL; n = 6; *p* = 0.007, F = 2.068) after 28 days (Figure 1C). These results suggested that our SGR protocol induced dry mouth. After we confirmed induction of dry mouth, we investigated whether dry mouth induced morphological changes in the taste buds and tongue epithelium. Both the basal layer and taste buds were found to be disrupted after SGR (Figure 1D). As shown in Figure 1E, impairment of parasympathetic innervation and loss of stem/progenitor cell differentiation were known to be important for morphological abnormalities in dry mouth (Figure 1E); subsequently, we analyzed the expression of epithelial, nerve innervation, and stem cell markers.

### 3.2. Comparison of Calcium Influx in Taste Bud Stimulation

**Figure 2:**
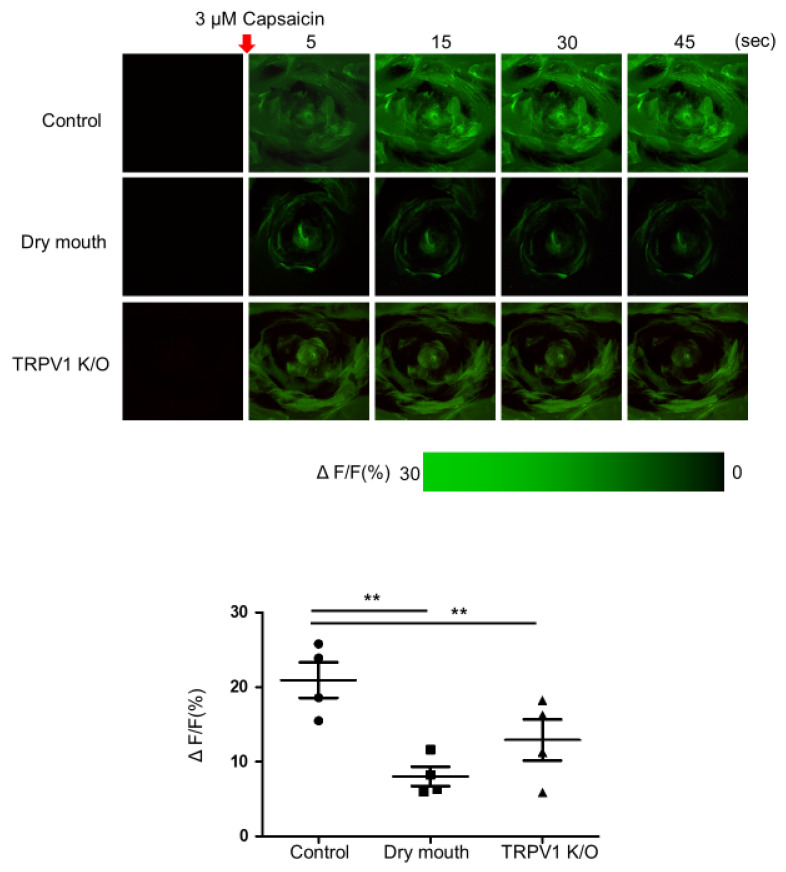
Live imaging of mouse tongue by two-photon microscope. To visualize the calcium reaction in the taste buds, 50 mM of an anionic calcium indicator (calcium green-1 dextran) was loaded onto the tongue dorsum using tweezer-type electrode (5 Vpp, 2 Hz, pulse width: 100 ms, duration: 10 s) and rinsed using PBS. After stimulation using 3 μM capsaicin, the live images of calcium activity were observed. Fluorescent images were captured at ~6 Hz with emissions at 525 ± 50 nm for anionic calcium indicator. ** *p* < 0.05.

Ionotropic receptors activation induced a voltage-gated calcium influx, and increased intracellular calcium operates a neurotransmitter release from a conventional chemical synapse [26,27]. Thus, we decided to stain the mouse tongue with anionic calcium indicator such as calcium green-1 dextran and observe the calcium influx. We performed the calcium dye staining using a tweezer-type electrode from the function generator several times and observed with a two-photon microscope for 45 s after we stimulated the mouse tongue using 3 μM capsaicin. Figure 2 and Supplement Video S1 (Video S1A–C) showed the live image of the calcium influx observation. The calcium reaction in the SGR group was significantly different from the control group. The tasty pore of the SGR group was darkened and fluorescence intensity was also low compared to the control group. The calcium influx after capsaicin stimulation was determined to be 20.95 ± 4.74% (n = 4) in the control group. The calcium level of the dry mouth group was decreased to 7.29 ± 3.63% (n = 4, *p* < 0.05, q = 8.64) compared to the control group. Interestingly, although the value was highly deviated, the calcium level of the TRPV1 K/O group was also lower than the control group at 10.68 ± 4.35% (n = 4, *p* < 0.05, q = 6.49).

### 3.3. Dry Mouth-Altered Keratin and NF Expression

**Figure 3:**
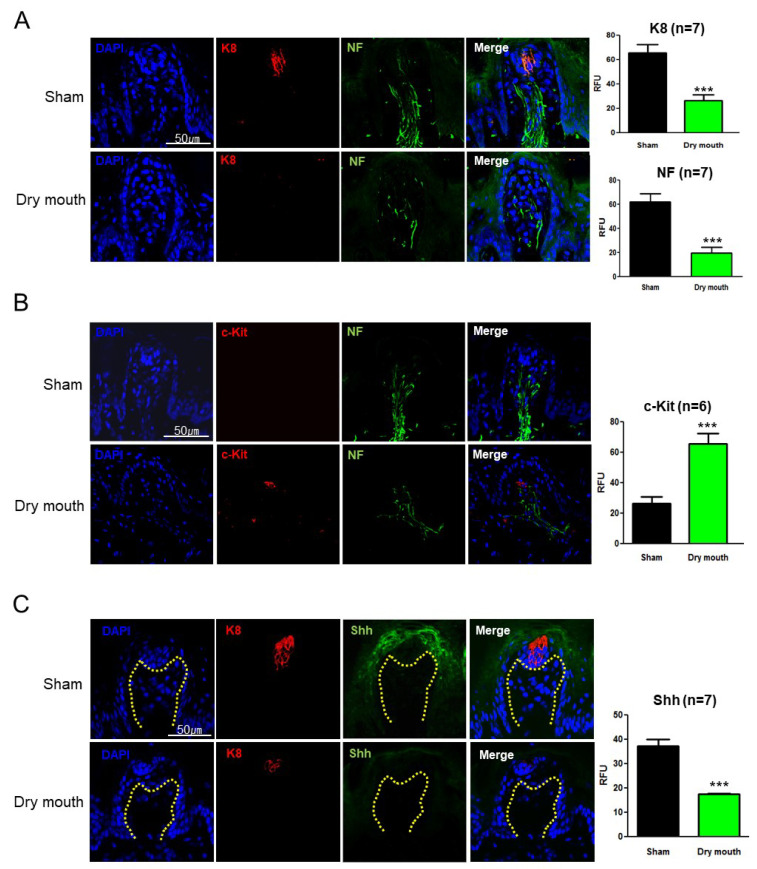

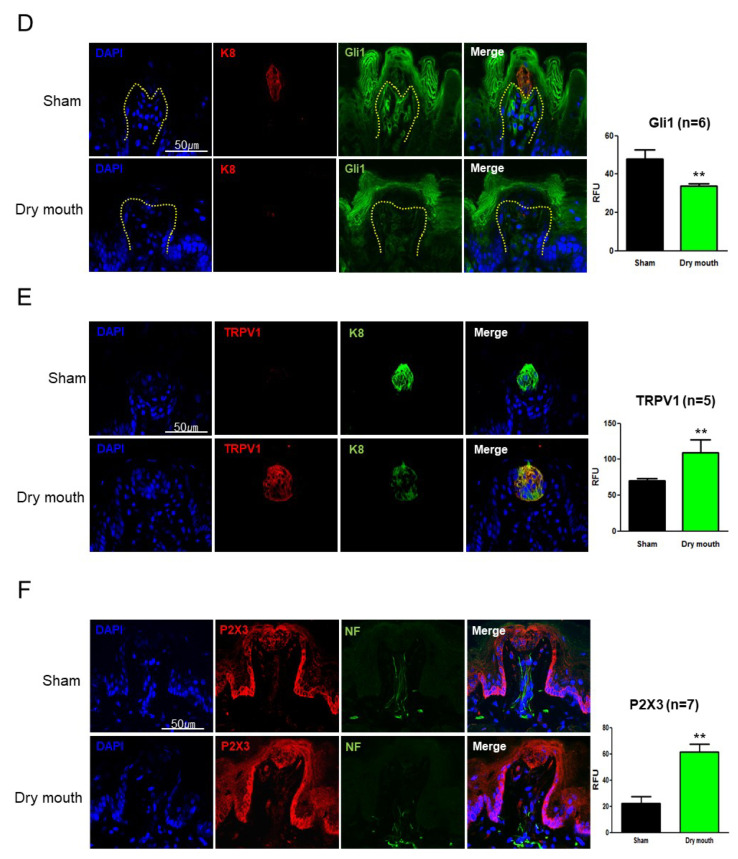
The expression of K8, NF, Shh, Gli1, c-Kit, TRPV1, and P2X3 in SGR mice. The expression of the taste bud epithelial cell markers, K8 and NF, considerably decreased in the dry mouth group. The c-Kit expression at the ends of NFs increased in the dry mouth group. Shh and Gli1 expression considerably decreased in the dry mouth group. However, TRPV1 and P2X3 (ligand-gated ion channel) expression was elevated in the dry mouth group. (**A**) Immunofluorescence staining of fungiform papillae and taste buds with K8-Alexa 657 (red) and NF-Alexa 488 (green). After DAPI mounting (blue), the samples were observed using confocal microscopy, and the intensity was measured and converted to relative fluorescence units (RFUs). The values have been provided in terms of mean ± standard error of mean (SEM). *** *p* < 0.001. The scale bar provided applies to all the images. (**B**) Immunofluorescence staining of fungiform papillae and taste buds with c-Kit-Alexa 657 (red) and NF-Alexa 488 (green). *** *p* < 0.001. (**C**) Immunofluorescence staining of fungiform papillae and taste buds with K8-Alexa 657 (red) and Shh-Alexa 488 (green). *** *p* < 0.001. The dashed lines demarcate the epithelial border. (**D**) Immunofluorescence staining of fungiform papillae and taste buds with K8-Alexa 657 (red) and Gli1-Alexa 488 (green). ** *p* < 0.05. (**E**) Immunofluorescence staining of fungiform papillae and taste buds with TRPV1-Alexa 657 (red) and K8-Alexa 488 (green). ** *p* < 0.05. (**F**) Immunofluorescence staining of fungiform papillae and taste buds with P2X3-Alexa 657 (red) and NF-Alexa 488 (green). ** *p* < 0.05.

To further assess the progression of taste bud disruption due to dry mouth syndrome, we analyzed K8 expression, which is essential for taste bud differentiation, and NF distribution, for studying innervation. The expression of K8, the epithelial cell marker on taste buds, in the dry mouth group was significantly lower than that in the sham group (*p* < 0.001, F = 1.804). NF retraction was also observed in the dry mouth group (*p* < 0.001, F = 1.840; Figure 3A). Furthermore, we studied hematopoietic stem cell factor (c-Kit), which is essential for taste bud development. The dry mouth group had significantly higher c-Kit expression than the sham group (*p* < 0.001, F = 3.308; Figure 3B). These results indicated that dry mouth induced the loss of epithelial characteristics, NF retraction, and c-Kit overexpression at the ends of NFs in the taste buds.

### 3.4. Dry Mouth-Induced NF Retraction Downregulated Shh and Gli1

Shh/Gli signaling is important for taste bud development [28,29]. We hypothesized that the elevated c-Kit expression was associated with Shh and Gli1 downregulation in taste buds. In each figure, we have drawn dashed lines between the taste buds and chorda tympani nerve, thereby marking the epithelial border. Shh expression was restricted to nerve endings and taste buds in the dry mouth group (*p* < 0.001, F = 3.767; Figure 3C). When K8 was downregulated, Shh expression and localization were entirely consistent with those in K8-positive cells. The Gli1 expression in the dry mouth group was significantly lower than that in the sham group (*p* = 0.007, F = 1.527; Figure 3D). These results suggested that the decreased keratin expression in dry mouth is associated with perturbed taste bud maintenance and disrupted Shh/Gli1 signaling.

### 3.5. TRPV1 Contributed to Taste Bud Development

TRPV1 serves as a pain receptor as well as a neurodevelopmental regulator of stem cells [21]. We hypothesized that TRPV1 is associated with taste bud maintenance in dry mouth and analyzed TRPV1 expression. TRPV1 expression in the dry mouth group was significantly higher than that in the sham group (*p* = 0.006, F = 11.76; Figure 3E). Similarly, the expression of P2X3, a ligand-gated ion channel, in the dry mouth group was also higher than that in the sham group (*p* = 0.001, F = 1.567; Figure 3F). These results suggested that overexpression of ligand-gated ion channel receptors such as TRPV1 and P2X3 provides feedback signals for decreasing innervations.

### 3.6. TRPV1 Affected Shh and c-Kit Expression

**Figure 4:**
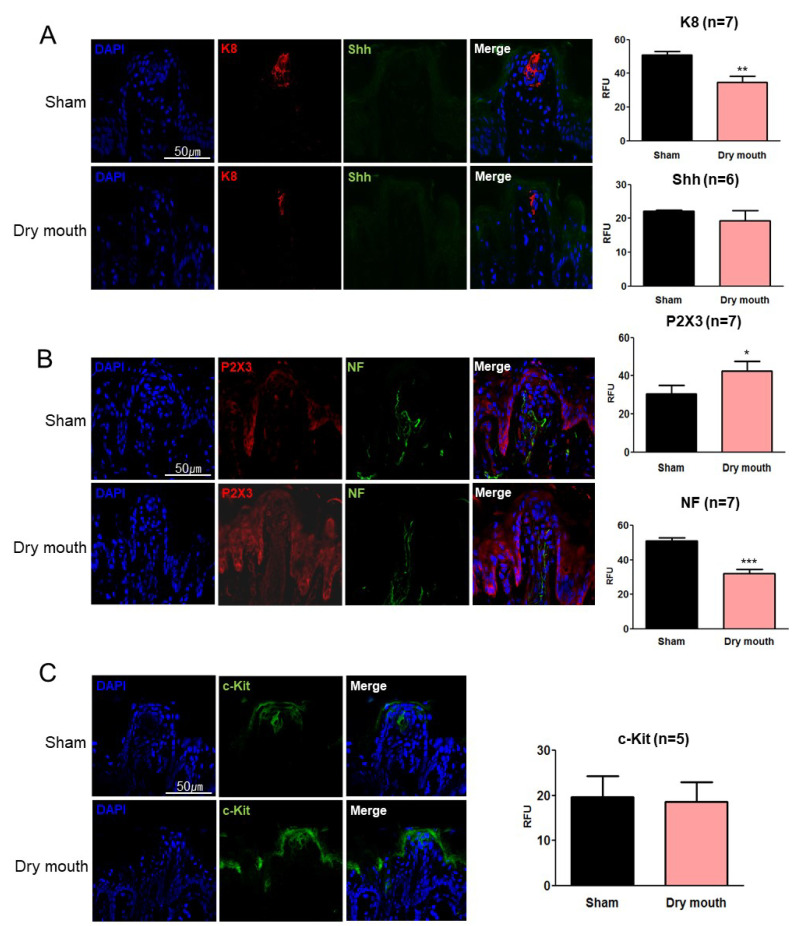
The expression of K8, Shh, P2X3, NF, and c-Kit in TRPV1 deficiency mice. Shh and c-Kit expression on the taste buds was influenced by TRPV1. NF and K8 expression decreased in the TRPV1-/- group, whereas P2X3 increased at the ends of NFs in the TRPV1-/- dry mouth and wild-type groups. Shh and c-Kit expression was not observed in the TRPV1-/- dry mouth group. (**A**) Immunofluorescence staining of fungiform papillae and taste buds with K8-Alexa 657 (red) and Shh-Alexa 488 (green). The values have been provided in terms of mean ± standard error of mean (SEM). ** *p* < 0.05. The scale bar provided applies to all the images. (**B**) Immunofluorescence staining of fungiform papillae and taste buds with P2X3-Alexa 657 (red) and NF (green). * *p* < 0.1. (**C**) Immunofluorescence staining of fungiform papillae and taste buds with c-Kit-Alexa 488 (green). *** *p* < 0.001.

To determine whether Shh expression was affected by TRPV1, we analyzed K8, Shh, P2X3, and c-Kit expression in both sham and SGR groups of TRPV1-/- mice. K8 expression was significantly downregulated in the TRPV1-/- dry mouth group (*p* = 0.003, F = 3.974; Figure 4A). Shh expression was downregulated in both the sham and dry mouth groups. These results suggested that TRPV1 mutation induced aberrant innervation and suppressed Shh. However, P2X3 expression was significantly upregulated (*p* = 0.018, F = 1.163) and NF expression was significantly downregulated (*p* < 0.001, F = 1.402) in TRPV1-/- mice in the dry mouth group (Figure 4B). This result indicated that P2X3 receptors were expressed in the dry mouth group regardless of TRPV1 expression, thereby causing NF retractions. Furthermore, c-Kit expression was significantly suppressed in TRPV1-/- mice in the dry mouth group (*p* = 0.438, F = 1.620; Figure 4C). This result also supported our hypothesis that TRPV1 affects taste bud development via Shh and c-Kit regulation.

## 4. Discussion

The taste organ of the tongue is an exquisite sensory system of papillae and resident taste buds, which is both skin, organ, and sensory nerve [30]. As shown in Figure 1E, the papillae are covered with a stratified squamous epithelium and attached to an underlying basal lamina. The taste buds in papilla epithelium are connected blood vessel, nerves, and Schwann cells in the papillary core. The tongue and taste organs receive innervation from the cranial ganglia. The tongue epithelium must turn over continuously; however, its unbalancing resulted in dry mouth. Hedgehog signaling is required for salivary branching morphogenesis [31] and is activated during functional regeneration of adult salivary glands after duct ligation [32]. Hedgehog signaling is stimulated by the binding of hedgehog ligands, such as Shh, with their receptor Patched (Ptch), which depresses Smoothened (Smo) to activate Gli transcription factors and, consequently, also activates hedgehog target genes, including Gli1 [12,28,33,34]. Numerous clinical studies have reported Shh downregulation in patients with gustatory dysfunction [11,29,35], with the most common symptom being dry mouth. Dry mouth is reported to be caused by burning mouth syndrome and is accompanied by pain due to overexpression of ligand-gated ion channels such as purinoceptors [12,36]. Among them, P2X is known to be a class of ligand-gated ion channel and it facilitates transmembrane Ca^2+^ and Na^+^ entry using ATP. TRPV1 is also a member of the TRPV group of transient receptor potential family of ion channels, and is a key element at the start of the pain pathway [37]. However, it has been reported to participate in pathways regulating and interacting with neurogenic signaling [38,39]. In this study, we investigated NF distribution, Shh/Gli1 expression, and the role of TRPV1 in dry mouth by using a mouse model of dry mouth syndrome. We confirmed the induction of dry mouth due to SGR, resulting in loss of body weight and salivation. We also observed morphological changes in taste bud fungiform papillae. Fungiform papillae contain gustatory receptor neurons in the nerves of taste buds, such as the chorda tympani, as well as three different types of sensory epithelial cells in taste buds; therefore, it was important to investigate the distribution of and signaling associated with NF. To investigate morphological changes induced by the epithelial and neuronal dysfunction of taste buds, we analyzed K8, NF, c-Kit, Shh, and Gli1 expression. In the dry mouth group, the expression of K8 and NF was found to be downregulated in the taste buds, while c-Kit expression was upregulated at the ends of NFs. Furthermore, the expression of Shh and Gli1 was downregulated in the dry mouth group. The K8-positive area in the taste buds overlapped with the Shh-positive area, which was accompanied by significant NF retraction in the dry mouth group. Thus, the current data indicated that innervation plays a key role in taste bud maintenance via Shh/Gli1 signaling.

c-Kit, a marker of progenitor/stem cells, was expressed at the ends of NFs in the dry mouth group. c-Kit expression also indicates the immature state of taste bud cells; however, it is reported to be influenced by TRPV family proteins through intracellular calcium concentration [Ca^2+^]*i* signaling [15,16,40]. Therefore, we investigated the TRPV1 and P2X3 responses in the dry mouth group. The results showed that, in the dry mouth group, K8 expression was significantly downregulated in the cells, whereas TRPV1 was robustly activated; moreover, P2X3 expression was upregulated at the ends of NFs. P2X3 belongs to the ATP-dependent purinoceptor family; it functions as a ligand-gated ion channel and transduces signals due to ATP-evoked nociception [41,42,43]. The results suggested that dry mouth induced the expression of nociceptors, such as P2X3, and triggered NF retraction, thereby activating TRPV1 in neuroepithelial cells in the taste buds.

To investigate the correlation between TRPV1 and Shh/Gli1, we analyzed K8, Shh, NF, P2X3, and c-Kit expression in TRPV1-deficient mice. A cone-shaped morphological alteration was observed in the fungiform papillae and the taste bud area was indiscernible in TRPV1-/- mice, suggesting that TRPV1 is involved in taste bud innervation [22,44,45]. Shh was rarely expressed even under normal conditions in TRPV1-/- mice, suggesting that innervation during taste bud development depended on functional TRPV1. Despite TRPV1 deficiency, K8 expression was significantly downregulated in the dry mouth group, which is probably due to several factors. However, K8 downregulation seemingly induced nerve degeneration due to dry mouth. Shh release from both nerves and epithelia is a crucial factor for taste bud maintenance; TRPV1 deficiency decreased the differentiation of neurons and neuroepithelial cells. Moreover, we found that P2X3 expression was upregulated in TRPV1-/- mice in the dry mouth group, suggesting that dry mouth can activate nociception independent of TRPV1. Stock’s study supports our findings well. Stock et al. reported that the knockout of TRPV1 expression resulted in a significant increase of postnatally proliferating cells compared to the wild-type. In addition, they demonstrated that TRPV1-knockout mice express stem cell genes such as Nestin or Sox2, and to a lesser extent, differentiation markers for astrocytes and neurons [44]. To our knowledge, the current study is the first to report that TRPV1 not only regulates the cell cycle via c-Kit but also regulates innervation during taste bud development via Shh/Gli1 signaling in dry mouth.

There is limited evidence to support the use of pilocarpine hydrochloride in the treatment of dry mouth. There is little evidence to support the use of other parasympathomimetic drugs in the treatment of dry mouth, but side effects can be problematic [46]. As a result, it seems that there is no treatment method that can replace chemical parasympathomimetics. Currently, regenerative strategies targeting salivary stem/progenitor cells provide promising options [47,48]. Nonetheless, the success of these strategies is constrained because the molecular basis of the maintenance of gustatory organs remains unclear. Recent research on the regulators of Shh/Gli1 [49,50] and Wnt/β-catenin [51,52] signaling has provided valuable insights into potential regenerative therapies. Our results indicated that TRPV1 promotes neural regeneration at the taste buds via Shh/Gli1 signaling, thus serving as a useful target for the clinical treatment of dry mouth.

## 5. Conclusions

Dry mouth is a condition that affects the quality of life, and it has a relationship with the poor regeneration and development of taste buds. This study demonstrated the contribution of P2X3/TRPV1 nociceptor on innervation via Shh/Gli signaling in taste buds, and identified the role of TRPV1 in dry mouth using an animal model. Dry mouth-induced NF retraction triggered the expression of nociceptors such as P2X3, and activated TRPV1 in neuroepithelial cells in the taste buds. However, TRPV1 deficiency resulted in retention of stemness in taste bud cells though a P2X3 increase. In conclusion, TRPV1 plays a crucial factor in neural regeneration via Shh/Gli signaling, which can be used as a clinical treatment target.

## Data Availability

The data presented in this study are openly available https://doi.org/10.5281/zenodo.7214193.

## Supplementary Materials

The following supporting information can be downloaded at: https://www.mdpi.com/article/10.3390/life13010075/s1, Figure S1: Genotyping PCR results of TRPV1 K/O mice. Figure S2: Scheme of calcium green loading for live imaging. Video S1: Live imaging of mouse tongue by two-photon microscope (A) Control; (B) Dry mouth; (C) TRPV1 K/O.

## Abbreviations

SGR: salivary gland resection; K8: keratin 8; NF: neurofilament; Shh: sonic hedgehog; Gli1: glioma-associated oncogene homolog 1; TRPV1: transient receptor potential vanilloid 1; P2X3: P2X purinoceptor 3; c-Kit: hematopoietic stem cell factor; OCT: optimal cutting temperature; RFU: relative fluorescence unit.
