# Supplementary material for: Role of Transient Receptor Potential Vanilloid 1 in Sonic Hedgehog-Dependent Taste Bud Differentiation
Source: Life (Basel). 2022 Dec 27;13(1):75. doi: 10.3390/life13010075 (PMC9862146; doi:10.3390/life13010075)
Supplement: Supplementary file 1 [file life-13-00075-s001.zip › life-2004421-supplementary.pdf]

Supplementary Figure S1: Genotyping PCR results of TRPV1 K/O mice.

The Jackson Laboratory

Return to Protocol Search

Stock Number: **003770**

Strain Name: B6.129X1-Trpv1^{tm1Jul}/J

Allele: Trpv1^{tm1Jul}

Protocol Name: Trpv1^{tm1Jul}-Alternate 1

Method: Standard PCR

Version: 1.2

Created: 19-May -2017 Updated: 24-May -2017

Expected Results:

Mutant = 176 bp
Heterozygote = 176 bp and 289 bp
Wild type = 289 bp

Gel Image

Image	Image file
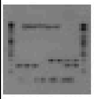	View

Protocol Primers

Primer	5' Label	Sequence 5' –> 3'	3' Label	Primer Type	Note
19922		TGG CTC ATA TTT GCC TTC AG		Wild type Forward	Reaction A
19923		CAG CCC TAG GAG TTG ATG GA		Common	Reaction A
oIMR1627		TAA AGC GCA TGC TCC AGA CT		Mutant Forward	Reaction A

#1 #2 #3 #4 #5 #6 #7 #8 #9 #10 #11 #12

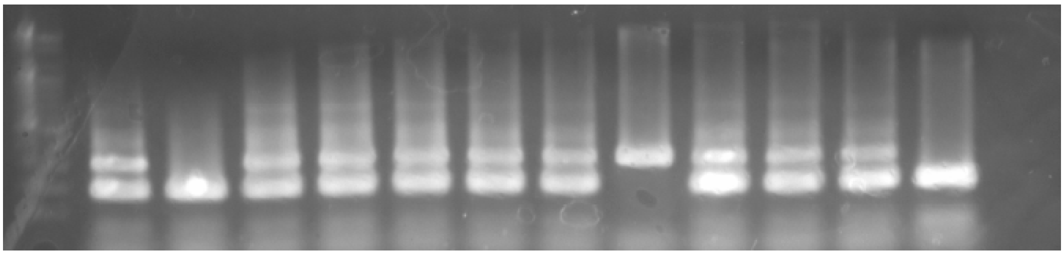

H M H H H H H W H H H M

Wild: 289bp
Heterozygote: 176 and 289bp
Mutant: 176bp

Supplementary Figure S2: Scheme of calcium green loading for live imaging.

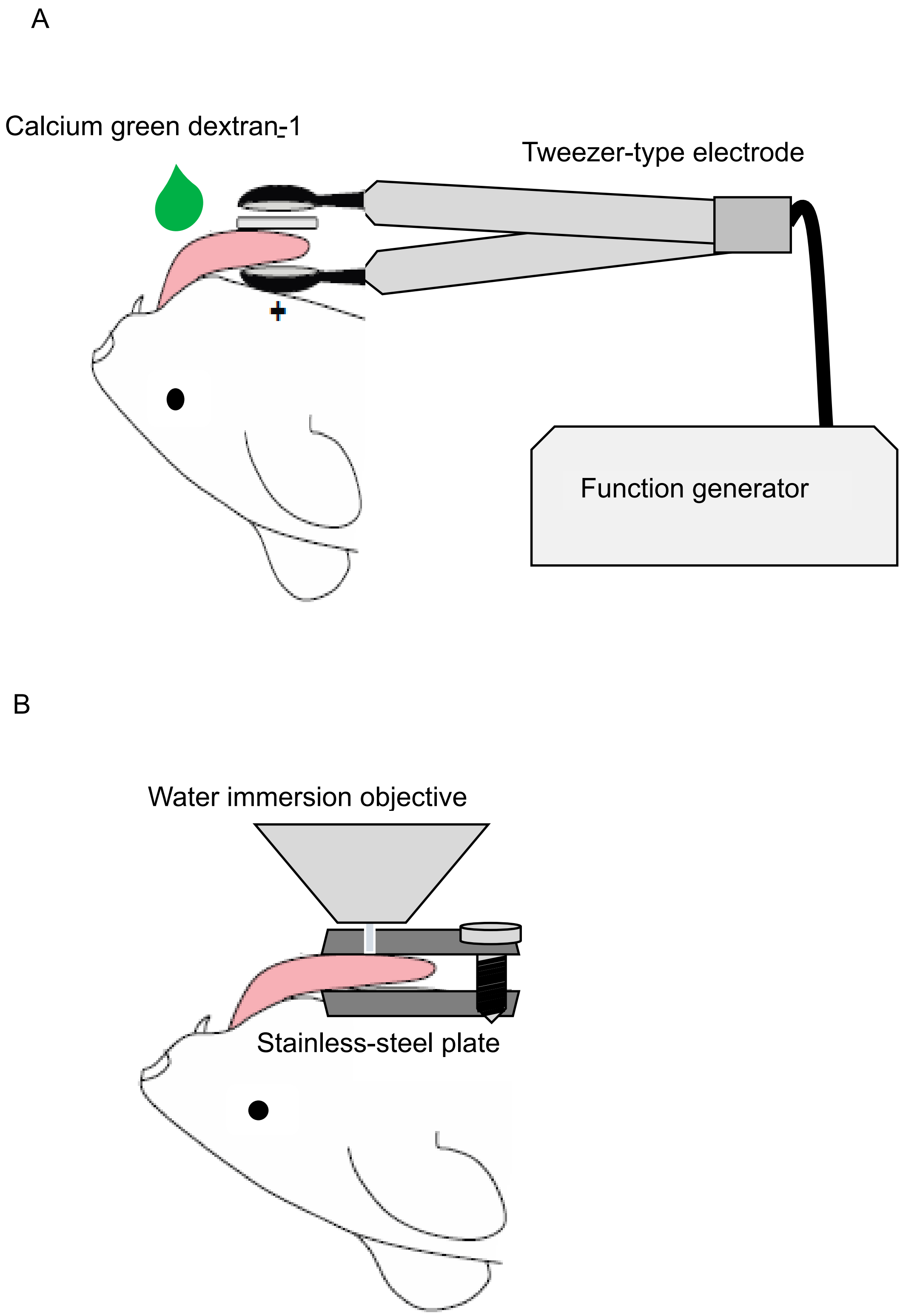

Video S1: Live imaging of mouse tongue by two-photon microscope (A) Control; (B) Dry mouth; (C) TRPV1 K/O (Attached separately as a video file.)
